# The occurrence of insectivores (Mammalia, Eulipotyphla) in Georgia from 1864 through to 2022

**DOI:** 10.3897/BDJ.11.e106256

**Published:** 2023-07-11

**Authors:** Andrei Kandaurov, Alexander K. Bukhnikashvili, Giorgi Sheklashvili, Ioseb Natradze

**Affiliations:** 1 Institute of Zoology, Ilia State University, Tbilisi, Georgia Institute of Zoology, Ilia State University Tbilisi Georgia

**Keywords:** Caucasus, Georgia, mammals, Eulipotyphla, biodiversity, species distribution, occurrence records, museum collection

## Abstract

**Background:**

Of the 108 species that occur in Georgia, ten species are insectivores belonging to the order Eulipotyphla. Forty percent of them are endemic to the Caucasus and sixty percent are endemic to the Middle East, including the Caucasus. Up to now, no comprehensive data on the distribution of insectivores in Georgia have been available.

The aggregated standardised data on the occurrence of small mammals can be applied to resource management, biogeography, ecological and systematic studies and to the planning of nature conservation efforts. Hereafter, the attempt to provide accumulated in one paper all known points of insectivores' occurrence in Georgia and make it available to researchers via the open repository GBIF is presented.

The dataset is based on both literature data from 30 published sources (251 records), collection vouchers from four main zoological collections containing vouchers from Georgia (415 records) and authors' fieldwork results (217 records). The occurrence points of the specimens stored in collections and museums have been extracted from museum voucher labels and museum journals.

**New information:**

All known sampling points of insectivores in Georgia are collected in one dataset for the first time. Our field surveillance data reach about 24.6% of the records. Most of our data collected since 2003 have not been published yet. About 28.4% of the records have been recovered from publications in Russian and Georgian languages and 47% of the dataset records are derived from collections.

## Introduction

In 1999, the Caucasus was designated as one of the 25 world biodiversity hotspots (Zazanashvili et al. 1999), with a high level of endemism and seventy percent of "its habitat diversity". The Caucasus is known for the number of relict endemic species (Tarkhnishvili 2014). Now, it is still one of the 36 Global Hotspots (Mittermeier et al. 2011), but the distribution of the species still needs investigation (Mumladze et al. 2019).

Of the ten species of insectivores occurring in Georgia, one species belongs to the family Erinaceidae, two species to the family Talpidae and seven species belong to the family Soricidae (Bukhnikashvili and Kandaurov 2002). One species, the Caucasus mole (*Talpacaucasica*), is endemic to the western part of Georgia, three shrew species are endemic to the Caucasus (*Sorexraddei*, *S.volnuchini*, *Neomysteres*), one mole (*Talpalevantis*) and one shrew (*S.satunini)* are endemic to the Caucasus and Minor Asia Peninsula and the hedgehog (*Erinaceusconcolor*) occurs in the Caucasus, Minor Asia Peninsula and the eastern coast of the Mediterranean Sea. The other three shrews (*Crociduraleucodon*, *C.suaveolens* and *Suncusetruscus*) are widespread species.

The representatives of the insectivorous mammals occurring in Georgia appeared in the general faunistic scientific publications for the first time at the end of the 18^th^ century (Güldenstädt 1791; Pallas 1811) and the knowledge collected during the 19^th^ century was summarised by G.Radde (Radde 1899). However, their distribution in Georgia has not yet been sufficiently studied. The first publications devoted to insectivores were published at the beginning of the 20^th^ century by K. Satunin (Satunin 1903, Satunin 1914, Satunin 1915).

A few of publications are dedicated to this group of mammals (Shidlovskiy 1953; Andghuladze 1960; Deparma 1961) between the works of K. Satunin and a large monograph of A. Tembotov and V. Sokolov (Sokolov and Tembotov 1989). Most of the distribution points can be found in general, ecological, systematic and faunal reviews (Janashvili 1953; Papava 1953; Enukidze 1958; Shidlovskiy 1958; Papava 1960; Janashvili 1963; Shidlovskiy 1964; Avaliani 1969a, Avaliani 1969b, Avaliani 1970, Avaliani 1973, Avaliani 1976; Shidlovskiy 2013). Some of the data can be extracted from publications devoted to the Caucasus or Eurasia and to widespread species which are recorded in Georgia (Ognev 1926, Ognev 1928; Deparma 1961; Pavlinov and Rossolimo 1987; Kandaurov et al. 1994).

Certain information about the distribution of insectivores in Georgia is given in Dzuev et al. (1972); Dzuev (1980), Dzuev (1981), Tembotova (1987) and Dzuev (1988). The book "Mammals Insectivorous. The Series Vertebrates of Caucasus" (Sokolov and Tembotov 1989) was published almost seventy years after K. Satunin's book "Mammals of the Caucasian Region (Chiroptera, Insectivora, Carnivora)" (Satunin 1915). This book mentions only one hundred and twelve records of all species of insectivores occurring in Georgia. However, many findings of insectivores in Georgia, known from the Simon Janashia State Museum of Georgia and more than one hundred records from the Catalogue of the Collection of the Institute of Zoology of the Georgian Academy of Sciences (Morgilevskaia 1989), are omitted. The data obtained by the parasitologists studied the parasites of small mammals, including insectivores (Matsaberidze 1967, Matsaberidze 1976; Kurashvili et al. 1977) and data presented in the early studies, devoted to the fauna study of particular regions of Georgia (small-scale research territories) (Andghuladze 1960; Avaliani 1969a, Avaliani 1969b, Avaliani 1976), were not used in this book.

Part of our field data were published in local issues and conferences proceedings (Kandaurov and Bukhnikashvili 1990; Bukhnikashvili and Kandaurov 1992; Eriashvili and Kandaurov 1992; Kandaurov 1992; Kandaurov 1997; Bukhnikashvili and Kandaurov 2001, Bukhnikashvili and Kandaurov 2002).

The situation changed after A. Bukhnikashvili published all available data on the distribution of small mammals in Georgia (Bukhnikashvili 2004). All of the sources above and all data known at that time, including information from the museum collections of the Zoological Institute of the Russian Academy of Sciences, the Zoological Museum of Moscow State University and the results of sampling performed by us, were combined and published.

Specimens collected in 450 locations in Georgia are stored at the Institute of Zoology of Ilia State University. Amongst them are specimens collected in 217 locations in Georgia in the course of recent research and specimens collected in 233 locations under the direction of M. Shidlovskiy previously (Morgilevskaia 1989; Kandaurov and Tskhadaya 2014; Kandaurov et al. 2015; Tskhadaia et al. 2019).

## General description

### Purpose

The data presented in the dataset combines all known insectivores sampling points in Georgia from the very beginning of small mammals collecting in the Caucasus until recent days. The records can be used for planning further efforts to investigate species ranges and ecological niche and modelling.

## Project description

### Funding

The data presented here were selected and extracted from the published sources and collections and the dataset was compiled in the framework of preparation work during the implementation of collaborative projects. The study was supported by the Shota Rustaveli National Foundation of Georgia (SRNSFG) [FR-19-2295] and by the US Defense Threat Reduction Agency (HDTRA 1-19-1-0044) "Preparation of the Atlas of Zoonotic Infections in South Caucasus supported by R. Lugar Center for Public Health Research, the National Center for Disease Control (NCDC, Georgia) and DTRA US".

## Sampling methods

### Study extent

The dataset contains information on 883 sampling point records (one species in a definite place, in a definite time) of the ten species. The occurrences were recorded between 1864 and 2022. The study area is Georgia, about 69700 km^2^. The occurrences are spread between 0 and 3021 metres above sea level.

The dataset can be divided by sources into three groups:

- 251 published sampling points (1886-1989);
- 415 occurrence points of samples stored in collections and museums (1864-2015) ;
- 217 points of the authors' field surveillance data – published and unpublished (1978-2022).

### Sampling description

Four types of records with known locations are retrieved from the following sources: the literature (published sources), the collection documentation (primary labels, individual labels and collection inventory documents) and our field observation results without precise coordinates, obtained without GPS usage and with coordinates obtained using GPS are combined in the presented dataset.

Data are taken from publications and documents that provide information for the first time on the occurrence of a particular species in a specific place in Georgia. Amongst the 30 publications, 18 are published in Georgian and 12 in Russian.

Field diaries and dissection journals were used for the authors' data that are not yet published. About 25% of all records in the dataset are presenting the authors' field observation data. Geographical latitude/longitude coordinates for 75% of them, or about 18.5% of all records in the dataset, were obtained using GPS.

Occurrences derived from the literature and collection vouchers were included in the dataset only if we were able to determine their coordinates. For samples without coordinates obtained from old museum collections, published sources and our field data before 2001, we did georeferencing using Google Earth. We have been able to find the nearest suitable geographic point in the cases when the vernacular names of sampling sites (i.e. names of villages, mountains, rivers etc.) and the habitat descriptions were provided in the publications or collection documents, such as collectors labels, museum journals, field diaries etc.

**Figure 1.**
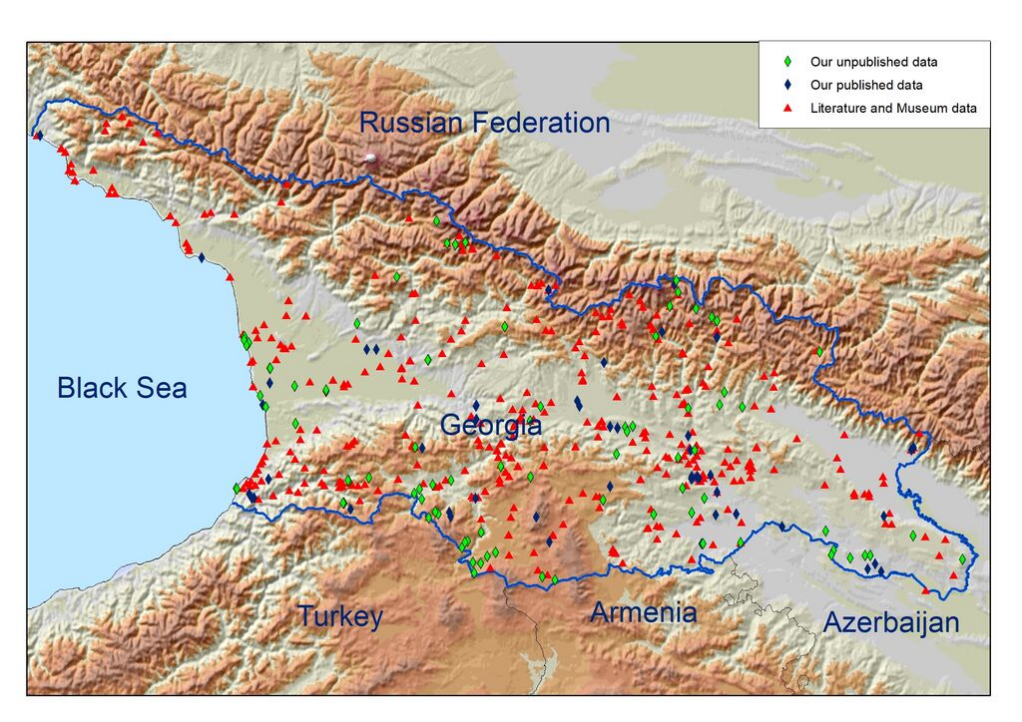
Records distribution across Georgia.

The coordinates are given in degree decimal format in the WGS84 system. The precision of the coordinates depends on the source. In the case of our field observation, using the GPS device, it is about 30-100 m. In the case of data from the literature and collection samples, we had an accuracy of about 800-1000 m. The spatial distribution of the insectivores' finding points within the limits of the study area is shown on the map (see Fig. 1).

### Quality control

All captured animals were determined at the species level using morphological criteria in the field (Gureev 1979; Kryštufek and Vohralik 2001; Zaitsev et al. 2014). Animal dissection and measurements are done following recent protocols (Barnett and Dutton 1995; Mills et al. 1995; Herbreteau et al. 2011). The total preparations, skulls and skins and tissue samples in alcohol are stored in the Institute of Zoology.

Each record in the dataset contains the following information: species name, municipality, locality name, known event date, coordinates, coordinate uncertainty in metres, coordinate precision, altitude, source of information and sources of georeference.

**basisOfRecord**: All records retrieved from published sources or from museum documents are marked as *MaterialCitation*. Records obtained as a result of our field observation are listed as *HumanObservation*.

**scientificName**: In the dataset, we followed the taxonomy presented in the third edition of the "Mammal Species of The World" (Wilson and Reeder 2005) available at the Smithsonian Institute (Washington, USA) site http://www.departments.bucknell.edu/biology/resources/msw3/ and used at the IUCN Red Data List website (IUCN 2022). For the species considered, the most recent novelties and corrections are not registered in the Official Registry of Zoological Nomenclature, ZooBank (https://zoobank.org). However, there is a reasonable opinion that the Lesser White-toothed Shrew occurring in Georgia belongs to the species *Crociduragueldenstaedtii* rather than *C.suaveolens* (Tez 2000;Burgin and He 2018; Ibiş et al. 2022). Consequently, after the final recognition of the separate species status of *Crociduragueldenstaedtii* for the Lesser White-toothed Shrew in Transcaucasia, it should be kept in mind that all records for *C.suaveolens* species should be considered as *Crociduragueldenstaedtii*. In addition, it cannot be excluded that, following the opinion of M. Shidlovskii, this species will be divided into two separate species in the future (Shidlovskiy 1953). Additionally, there is an opinion that the Caucasian mole (*Talpacaucasica*) that inhabits Georgia should be correctly referred to as the Ognev mole species (*T.ognevi*) (Bannikova et al. 2015; Kryštufek and Motokawa 2018) and the Levant mole (*Talpalevantis*) should be renamed as the Transcaucasian mole (*T.transcaucasica*) (Burgin et al. 2020). However, the boundaries of the ranges of these closely-related species on the territory of Georgia have not been defined. The territory of Georgia is located on the southern and northern macroslopes of the Greater Caucasus. Therefore, the occurrence of both species here cannot be ruled out without an additional study of the material from Georgia. These circumstances compel us to use, in the dataset, the system which is established and readily available online (Wilson and Reeder 2005). The number of records for each species is shown in Table 1.

**eventDate**: The date values for most records (about 70%) are presented as a date of capture if available; however, for some museum samples and 99% of records, retrieved from published sources, we have no date. In this case, the time of the occurrence can be roughly estimated as the year before which the species was observed there using the year of the oldest publication in which these sampling points are mentioned. About 2.3% of the records retrieved from the collection of the Institute of Zoology of Ilia State University have no dates. However, for 99.5% of the records, at least the year of the observation is known. Amongst the records retrieved from the Janashia State National Museum of Georgia no date is available for about 15%, but for about 93% of the records, the year of the observation is known. Besides, we do not have dates for 17% of the records obtained from the museum collection of the Zoological Institute of the Russian Academy of Sciences and for about 11% of the records retrieved from the Zoological Museum of Moscow State University.

## Geographic coverage

### Description

**Figure 2.**
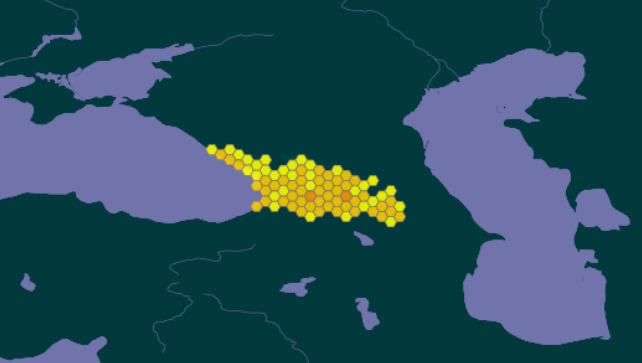
The geographical distribution of the occurrence records within the Caucasus Isthmus. DOI https://doi.org/10.15468/fb3akq. The difference in the colourings of the points is caused by the generalisation of the map. It reflects the number of species marked at a given location. The more species fall into one hexagon, the darker the shade of yellow in its colouring.

Description: Georgia is situated in the western part of the Caucasus isthmus on the Black Sea coast. The area of Georgia is about 69700 km^2^. The geographical distribution of the occurrence records within the Caucasus is shown in Map 2 (Fig. 2i). Georgia occupies the south macroslope of the Greater Caucasus range, the western part of the intermountain Transcaucasian depression divided by the Likhi Ridge into the Colchis Lowland in the west of the country and the Kura River Valley in the east of the country, the western part of mountain ranges of the Lesser Caucasus and the northern extremity of the Middle East Uplands, the Armenian Highlands, to the south from the Lesser Caucasus. From the climatic and landscape standpoint, the territory of Georgia is quite uneven. About 20% of Georgia’s territory is situated at an altitude higher than 2000 m above sea level (World Bank Group; Asian Development Bank 2021). About 40% of the territory of the country is covered by different types of forests.

According to the maps of the biogeographical regions in Europe (Cervellini et al. 2020), the Alpine, Black Sea, Anatolian and Steppic regions reach into the territory of Georgia. All these regions belong to the Eurasian or Palearctic realm according to the updated "An updated Wallace's zoogeographic regions of the World" (Olson et al. 2001; Holt et al. 2013). However, a certain part of Georgian territory, namely the northern slopes of Trialeti Ridge and part of the southern slopes of the Great Caucasus in eastern Georgia, is covered with forest areas with communities including Colchis elements of the Black Sea Region, East European elements belonging to Alpine Region, Middle East elements of the Anatolian Region and elements of the Steppic fauna. Therefore, these areas cannot be referred to as the above-named biogeographic regions with certainty. It is rather difficult to outline the correct border between the faunistic regions represented throughout Georgia due to the mutual penetration of species between them. The complicated, sometimes mosaic, spatial structure of biological communities representing different biogeographic regions is specific to Georgia, as well as to the entire Caucasus. A refuge of Tertiary flora is situated in Georgia, the Colchis refugium in the catchment basin of the Black Sea (Tarkhnishvili et al. 2011).

The most northern occurrence has the following coordinates: 43.5209N and 40.6382E, the most western occurrence - 43.388N and 40.0436E, the eastern occurrence - 41.2643N and 46.6313E and the most southern occurrence - 41.1626N and 43.805E.

### Coordinates

41.1626N and 43.5209N Latitude; 40.0436E and 46.631E Longitude.

## Taxonomic coverage

### Description

The dataset completely covers the fauna of insectivores that occur in Georgia. According to the contemporary standpoint in the taxonomy (Wilson and Mittermeier 2018), the fauna of insectivores in Georgia consists of three families belonging to one order Eulipotyphla. Earlier, there were accepted two separate orders Soricomorpha and Erinaceomorpha (Wilson and Reeder 2005). Only one species of the genus *Erinaceus*, belonging to the family Erinaceidae, occurs in Georgia. Two species of moles belonging to the genus *Talpa* are included in the family Talpidae. In the Soricidae family, there are two subfamilies, Soricinae, with two genera, *Sorex* (with three species) and *Neomys* (with one species) and Crocidurinae with two genera, *Crocidura* (two species) and *Suncus* (one species). For more details, see Table "Taxa included".

### Taxa included

**Table:** 

Rank	Scientific Name	Common Name
kingdom	Animalia	Animals
subkingdom	Eumetazoa	Eumetazoan
phylum	Chordata	Chordates
class	Mammalia	Mammals
subclass	Theria	True Mammals
order	Eulipotyphla	Eulipotyphlans
family	Erinaceidae	Hedgehogs and Gymnures
subfamily	Erinaceinae	Hedgehogs
family	Soricidae	Shrews
subfamily	Soricinae	Red-toothed Shrews
subfamily	Crocidurinae	White-toothed Shrews
family	Talpidae	Talpids
subfamily	Talpinae	Old World Moles

## Temporal coverage

### Notes

All sampling points of insectivores in Georgia included in the dataset were obtained from 1864 through to 2022. The records can be divided by the time of finding into three groups:

- 1864-1920 – 97 records – about 11% of the total number of records;
- 1921-1991 – 607 records – 68.7% of all records;
- 1992-2022 – 204 records – 23.1% of all records.

## Usage licence

### Usage licence

Other

### IP rights notes

CC BY-NC 4.0

## Data resources

### Data package title

The insectivores of Georgia

### Resource link

https://www.gbif.org/dataset/4eb9162e-6336-4fc8-abc6-0948270d2387

### Alternative identifiers

https://cloud.gbif.org/eca/resource?r=geo_invo

### Number of data sets

1

### Data set 1.

#### Data set name

Theinsectivoresofgeorgia

#### Data format

Darwin Core Archive

#### Data format version

V.1.2

#### Description

The dataset contains information on 883 sampling points records of the ten species of insectivorous mammals in Georgia. The occurrences were recorded between 1864 and 2022 (Kandaurov et al. 2023). Each record in the dataset contains the following information: species name, municipality, locality name, known event date, coordinates, coordinate uncertainty in metres, coordinate precision, altitude, source of information and sources of georeference.

**Data set 1.** 

Column label	Column description
occurrenceID	Unique identifier of a record.
Kingdom	The full scientific name of the kingdom in which the taxon is classified.
Phylum	The full scientific name of the phylum in which the taxon is classified.
Class	The full scientific name of the class in which the taxon is classified.
Order	The full scientific name of the order in which the taxon is classified.
Family	The full scientific name of the family in which the taxon is classified.
scientificName	Species' full scientific (Latin) name including authorship and year.
municipality	The full, unabbreviated name of the next smaller administrative region than county (city, municipality etc.) in which the Location occurs.
Locality	The specific description of the place of collection.
eventDate	Collection event date.
countryCode	Standard ISO 3166-1-alpha-2 country code.
decimalLatitude	The geographic latitude (in decimal degrees).
decimalLongitude	The geographic longitude (in decimal degrees).
geodeticDatum	Geographic coordinates reference system EPSG.
coordinateUncertaintyInMetres	Coordinate measurement accuracy (metres in the case of GPS recordings, blank - if manually georeferenced).
coordinatePrecision	A decimal representation of the precision of the coordinates given in the decimalLatitude and decimalLongitude.
minimumElevationInMetres	Minimum elevation above sea level.
maximumElevationInMetres	Maximum elevation above sea level.
associatedReferences	Source for the particular record.
georeferenceSources	The system used during the georeferencing.
basisOfRecord	The specific nature of the data record.
institutionCode	The code of the institution where data is stored.
collectionCode	The code of the collection.

## Figures and Tables

**Table 1.** Database content by species.

	Species	Endemic to the Caucasus	Records Number	Published Sources	Collections	Our data
1	*Erinaceusconcolor*		112	41	37	34
2	*Talpacaucasica*	Y	117	47	65	5
3	*Talpalevantis*		82	38	32	12
4	*Neomysteres*	Y	45	14	25	6
5	*Sorexraddei*	Y	57	17	26	14
6	*Sorexsatunini*		62	15	29	18
7	*Sorexvolnuchini*	Y	36	10	21	5
8	*Crociduraleucodon*		78	16	30	32
9	*Crocidurasuaveolens*		289	52	149	88
10	*Suncusetruscus*		5	1	1	3
	**Total**	**4**	**883**	**251**	**415**	**217**
